# Highly Expressing *SCARA5* Promotes Proliferation and Migration of Esophageal Squamous Cell Carcinoma

**DOI:** 10.1155/2022/2555647

**Published:** 2022-06-17

**Authors:** Kawuli Jumai, Tangjuan Zhang, Bingzhang Qiao, Julaiti Ainiwaer, Haiping Zhang, Zhichao Hou, Idris Awut, Madinyat Niyaz, Liwei Zhang, Ilyar Sheyhidin

**Affiliations:** ^1^Department of Thoracic Surgery, First Affiliated Hospital of Xinjiang Medical University, Urumqi, Xinjiang 830054, China; ^2^State Key Laboratory of Pathogenesis, Prevention, Treatment of High Incidence Diseases in Central Asia, Urumqi, Xinjiang 830054, China; ^3^Department of Emergency, First Affiliated Hospital of Zhengzhou University, Zhengzhou, Henan 450052, China; ^4^Department of Thoracic Surgery, First Affiliated Hospital of Zhengzhou University, Zhengzhou, Henan 450052, China; ^5^Clinical Medical Research Institute, First Affiliated Hospital of Xinjiang Medical University, Urumqi, Xinjiang 830054, China

## Abstract

**Background:**

Thrombospondin type 1 domain-containing 7A (*THSD7A*) was reported to play a procancer role in esophageal squamous cell carcinoma (ESCC). The aim of the study was to screen the downstream functional genes of *THSD7A* and explore their functions in ESCC, based on the reported research into *THSD7A* function and on gene microarrays.

**Methods:**

We adopted quantitative reverse-transcription polymerase chain reaction (qRT-PCR) and Celigo high-content screening (HCS) technology to screen the downstream genes of *THSD7A*. The expression level of target genes was examined by PCR, western blot, and immunohistochemistry (IHC). The effects of these target genes on ESCC malignant biological behavior were performed *in vivo* and *in vitro*. The Kaplan-Meier (K-M) survival analysis and Cox regression were used to analyze the prognostic significance of target genes in ESCC patients. Experiments in the literature on liver cancer (LC) were repeated to verify the functions of these genes in different tumors. We further explored the cancer-promoting mechanism of target genes in ESCC by sequencing of the genes' exons.

**Results:**

Scavenger receptor class A member 5 (SCARA5) was proved to be the downstream driving gene of *THSD7A*. SCARA5 promoted cell proliferation and migration but inhibited apoptosis in ESCC. IHC results confirmed that *SCARA5* expression in ESCC exceeded that in normal tissues. The K-M survival analysis indicated that *SCARA5* expression quantity was not related to prognosis, but tumor volume and T classification were both the independent prognostic factors. Repetition of experiments in LC in the literature confirmed that *SCARA5* had exactly opposite functions in EC and LC.

**Conclusion:**

*SCARA5* was related to the development and occurrence of ESCC. Our findings suggested that it was a potentially diagnostic individualized therapeutic target for ESCC in the future and that its application could possibly be combined with that of upstream THSD7A gene.

## 1. Introduction

Esophageal cancer (EC) has been reported as the 8th most common malignancy, as well as the 6th most common cause of cancer deaths in the world [1]. Every year, approximately 350,000 new cases of EC are diagnosed globally, and 200,000 people die of EC annually [2]. EC morbidity and mortality rates in China are both the highest worldwide, accounting for more than 50% of worldwide toll. China also has the highest incidence of squamous cell cancer, which comprises >90% of its EC cases [3]. Therefore, it is important to explore candidate genes that could have significance to the clinical diagnosis, as well as the treatment of esophageal squamous cell carcinoma (ESCC) at the molecular level [4]. Our previous study found that the thrombospondin type 1 domain-containing 7A (*THSD7A*) gene plays a procancer role in ESCC and participates in important signaling pathways, including mammalian target of rapamycin (mTOR). However, whether there are functional target genes downstream of *THSD7A* in ESCC has not been reported. We found that scavenger receptor class A member 5(*SCARA5*), located on chromosome 8p21.1, is the downstream driving gene of *THSD7A* in ESCC [5]. *SCARA5* has significant class A scavenger receptor characteristics [6]. Studies on SCARA5 are mainly focused on tumors, inflammatory responses, and cardiovascular disease (CVD) [7–14]. SCARA5 expression is significantly downregulated in various tumor tissues including liver cancer (LC), rectal cancer, and glioblastoma [15, 16] and might affect cell proliferation and invasion abilities by the focal adhesion kinase (FAK) signaling pathway and epithelial-mesenchymal transition (EMT) [5, 17–20]. *SCARA5* has been proved to be related to tumor occurrence and development. It is a tumor suppressor in various tumors, including LC and breast cancer (BC). Tumor-related studies have shown the clinical application and research prospects of this gene, suggesting that it might play the important role in the treatment of tumors. However, the expression level of *SCARA5* in ESCC and the gene's effect on the occurrence and development of this disease have not yet been reported. Based on the abovementioned findings, quantitative reverse-transcription polymerase chain reaction (qRT-PCR), western blot, Celigo high-content screening (HCS), exon sequencing, and functional experiments were used to screen and verify multiple functional downstream genes of *THSD7A*, including *SCARA5*. Through HCS experiments, we determined that *SCARA5*, as the downstream driving gene of *THSD7A*, has a critical role in promoting ESCC. The combination of IHC with clinicopathological and prognostic analyses revealed the significantly enhancing *SCARA5* expression in cancer tissues compared to the adjacent. Our repetition of experiments mentioned in the previous studies confirmed that *SCARA5* functioned differently in different tumors. We used exon sequencing and wild-type (WT) gene overexpression to rule out the possibility of functional differences caused by mutations. These experiments laid a foundation for further study of *SCARA5*.

## 2. Materials and Methods

### 2.1. Patients and Samples

A tissue microarray for IHC analysis of *SCARA5* was provided by Shanghai Outdo Biotech Co., Ltd (Shanghai, China). The microarray contained tissues from 100 ESCC cases and 80 paired normal adjacent tissues. The 100 patients included 74 men and 26 women. Patients' median age was 68 years (48–82 years). Patient's tumor staging and grade scores followed the seventh American Joint Committee on Cancer (AJCC)/Union for International Cancer Control (UICC) and tumor, node, and metastasis (TNM) classification of EC. All of the clinical and pathological parameter data for the analysis were obtainable for the 70 of 100 cases of ESCC. Of the other 30 cases, 14 had no gross information available, while the other 16 cases had no tumor volume information available. Patients did not receive any therapy before esophagectomy.

### 2.2. Cell Culture

Three human EC cell lines were included. Eca109 and TE-1 were obtained from the Shanghai Cell Bank of Chinese Academy of Sciences, and EC9706 was from China Center for Type Culture Collection (Wuhan, China). All cells were maintained in Dulbecco's Modified Eagle Medium (DMEM) containing 10% fetal bovine serum (FBS) (Gibco, Karlsruhe, Germany) containing 1% penicillin and streptomycin solution. The cells were grown at 37°C, 5% CO_2_, and humidified atmosphere.

### 2.3. Transfection of shRNA and sgRNA Vectors

Short-hairpin and single-guide ribonucleic acid (shRNA and sgRNA) sequences against target genes and scramble sequences and lentiviral vectors of target genes containing hU6, ubiquitin, multiple cloning site (MCS), internal ribosome entry site (IRES), and enhanced green fluorescent protein (eGFP) were both constructed and synthesized by GeneChem (Shanghai, China). We transfected the ESCC cells (5 × 10^4^/ml) with lentivirus-mediated target gene shRNA, sgRNA, and negative control. The cells were collected after transduction for 72 h and then used for qRT-PCR and western blot analysis.

### 2.4. Quantitative Reverse-Transcription PCR (qRT-PCR)

We isolated total RNA from samples of Eca109, EC9706, and TE-1 cells using TRIzol reagent (Invitrogen, Carlsbad, California, USA). We detected RNA purity and concentration using NanoDrop 1000 spectrophotometer (Thermo Fisher Scientific, Waltham, MA, USA). RNA was reversely transcribed into complementary deoxyribonucleic acid (cDNA) using a PrimeScript RT-PCR kit (TaKaRa, Dalian, China). We used an IQ5 RT-PCR system (Bio-Rad, Laboratories, Hercules, CA, USA) with SYBR Select Master Mix (Boster, Wuhan, China) to conduct qRT-PCR per manufacturers' instructions. Glyceraldehyde 3-phosphate dehydrogenase (GAPDH) was used as an internal reference for normalization. We evaluated the relative expression of target gene messenger ribonucleic acid (mRNA) via the 2-*ΔΔ*Ct method. The primers were shown in Table 1S.

### 2.5. Immunohistochemistry (IHC)

Subsequently, we washed the samples twice in xylene (5 min per wash); then with graduate ethanol, 95%, 70%, 50%, and 30%; and then in phosphate-buffered saline (PBS) in distilled water for 5 min on a shaker, step by step. We used 3% hydrogen peroxide to inactivate endogenous peroxidase. For the next step, the samples were heated in sodium citrate buffer (pH 6.0) in a microwave oven maintaining at 95°C for 15 min to retrieve the antigen. We incubated the tissue sections at 4°C, overnight, with rabbit anti-*SCARA5* primary antibody (1 : 200; Abcam, Cambridge, UK). After washing for three times with PBS, we added biotin-labeled secondary antibody (ZSGB-BIO, Beijing, China) and incubated for 15 min. After another wash, we used 3,3′-diaminobenzidine (DAB) to visualize immunoreactivity and hematoxylin for counterstaining. Staining was scored by the percentage (0, <5%; 1, 5-25%; 2, 26-50%; and 3, >50%) and positive staining intensity (0, none; 1, weak; 2, moderate; and 3, strong). The patients were divided into two, the low expression group (total score, ≤6) and the high expression group (total score, 6-9), according to the total score.

### 2.6. Western Blotting Assay (WB)

We used lysing buffer to process cells 72 h after transfection and determined protein concentrations using a BCA Protein Assay Kit (Beyotime Biotechnology, Shanghai, China). We separated 20 *μ*g of protein via 10% sodium dodecyl sulfate polyacrylamide gel electrophoresis (SDS-PAGE) and transferred it to polyvinylidene fluoride (PVDF) membranes at 100 V for 120 min. The membranes were blocked for 60 min at room temperature in blocking solution, probed overnight with mouse anti-GFP antibody (1 : 2000 dilution) and anti-GAPDH antibody (1 : 5000 dilution; both from Santa Cruz, Dallas, Texas, USA) at 4°C, and then incubated with secondary goat anti-mouse immunoglobulin G (IgG) antibody (Santa Cruz) for 120 min. We quantified bands and normalized protein intensity using Quantity One software (Bio-Rad).

### 2.7. Cell Cycle and Apoptosis Assay

Flow cytometry (FCM) was used to analyze cell cycle progression and apoptosis. For cell cycle analysis, we seeded cells (3 × 10^5^/well) into six-well plates and then cultured for 48 h. The cells were fixed in 75% cold ethanol and preserved at -20°C overnight. We washed cells, incubated them with PBS containing RNase (50 *μ*g/ml) for 1 h at 4°C, and stained them with propidium iodide (PI; 5 *μ*l, 1 mg/ml). We measured the distribution of G0/G1, S, and G2/M stages. Annexin V-adenomatous polyposis coli (APC) apoptosis detection kit (88-8007; eBioscience, Antibodies, San Diego, CA, USA) was used for apoptotic analysis. After washing cells twice with precooling PBS, 5 *μ*l Annexin V in 100 *μ*l binding buffer was added. The cells were then stained with 0.4 *μ*l of PI (10 mg/ml). A Cytomics FC500 FCM system was used to detect cells (Guava easyCyte HT; MilliporeSigma, Burlington, MA, USA).

### 2.8. Cell Invasion Assay

The cells (5 × 105/well) were seeded onto a fibronectin-coated polycarbonate membrane insert in a Transwell chamber with 8 *μ*m pores (BD Bioscience, Franklin Lakes, NJ, USA). We used 100 *μ*l serum-free media to culture the cells in the upper chamber and filled the lower chamber with 600 *μ*l DMEM containing 10% FBS. After culturing them for 10 h at 37°C in 5% CO2, we fixed the cells with methanol for 20 min and stained them with Giemsa staining solution (AR-0752; Ding Guo Biotechnology, Beijing, China) for 30 min. The number of invading cells was counted under an ECLIPSE TS100 microscope (Nikon, Tokyo, Japan). We counted five random fields per well and took the mean value.

### 2.9. Cell Wound Healing Assay

The cells were cultured and seeded in DMEM (10-013-CVR; Corning, Inc., Corning, NY, USA) serum-free medium at 96-well plates. We created a central linear using a 96-well mechanical floating pintool (VP408FH; V&P Scientific, San Diego, CA, USA). Images were obtained with fluorescent microscope (Olympus, Tokyo, Japan) at 0, 8, and 24 h. We measured scratch width and migration distance and then calculated mobility.

### 2.10. Colony Formation Assay

600 cells per well were inoculated in six-well plates, and then, the culture medium was replaced every 3 days. After 15 days growth, we fixed the cells with 4% paraformaldehyde (National Pharmaceutical, Shanghai, China) and stained them with Giemsa staining solution. The number of clones was counted manually.

### 2.11. MTT Assay

The cells were placed in a 96-well plate with a density of 2 × 10^3^ cell/well. At 0, 24, 48, 72, 96, and 120 h after transfection, we treated them with 20 *μ*l (5 g/l) methyl thiazolyl tetrazolium (MTT) (JT343; GenView Scientific, El Monte, CA, USA) at 37°C. After 4 h, remove the medium and replace it with dimethylsulfoxide (DMSO, 100 *μ*l/well). Absorbance (490 nm) was measured using a multiwall-plate reader (Bio-Rad).

### 2.12. Xenografted Athymic Nude-Mouse Model Assay

Animal experimental protocol in this study was in accordance with the *Guide for the Care and Use of Laboratory Animals* and received approval from the Animal Ethics Committee of the First Affiliated Hospital of Xinjiang Medical University, Urumqi, China. We constructed an Eca109-xenografted mouse model. We divided 20 BALB/c nude female mice, 5 weeks, weight 12.6–13.4 g, into two groups, experimental and control. The Eca109 cells were infected with lentiviral shRNA and the control, which were gained after serial subcultivation and purification. Then, we subcutaneously injected the infected Eca109 cells into the right axillary regions of the mice. After 30 days of feeding and observation, euthanasia was performed, and the tumors were collected and weighed.

### 2.13. Sequencing of *SCARA5* Gene Exons

Eca109, EC9706, and TE-1 were sent to Shanghai Biotechnology Sequencing Company (Shanghai, China) for exon sequencing. Primers were designed for nine exons of *SCARA5*, and then, four pairs of primers were designed for exon 9. After cell resuscitation, we extracted RNA for PCR amplification and subjected the amplified products to electrophoresis. After electrophoretic bands were obtained, we sequenced the exons and compared the results with the sequence of wild-type (WT) *SCARA5*.

### 2.14. High-Content Screening (HCS)

We cultured Eca109 cells in the knockdown (KD) and control groups of 11 target genes on 96-well plates. The cells were transfected with lentiviruses that had GFP fluorescent labels (GeneChem). After 72 h, when the fluorescence expression rate reached 80%, we reinoculated the cells into 96-well plates (2000 cell/well, 100 *μ*l/well). Celigo imaging cytometer (Nexcelom Bioscience, Lawrence, USA) was used to identify fluorescent cells, and we took photos to calculate the number of cells. After 120 h of continuous photography, we drew the cell growth curve and analyzed cell proliferation. Each group had three multiple wells.

### 2.15. Statistical Analysis

We conducted statistical analysis using SPSS software version 19.0 (IBM Corp., Armonk, NJ, USA). Pearson's chi-square and/or Fisher's exact tests were used for the correlation between *SCARA5* expression and patients' clinicopathological characteristics. *t*-test was used to calculate discrepancies between groups. On the basis of a log-rank test and Cox's proportional hazard regression model, a Kaplan-Meier (K-M) survival curve was used to analyze the remarkable between-patient differences in prognosis. Values were expressed as means ± standard deviations (SDs). A *P* < 0.05 value of two-sided was considered as significant level.

## 3. Results

### 3.1. Screening the Downstream Genes of *THSD7A*

In our previous study, we reported that THSD7A plays an important role in ESCC. 37 candidate genes listed in Table 1 were revealed after we compared the results from the Affymetrix human GeneChipPrimoView (Affymetrix, Santa Clara, CA, USA) and PubMed database. All of these genes are rarely reported in esophageal cancer.

### 3.2. Detecting the Downregulation Rate of the Candidate Genes via shRNA-*THSD7A*

**Figure 1:**
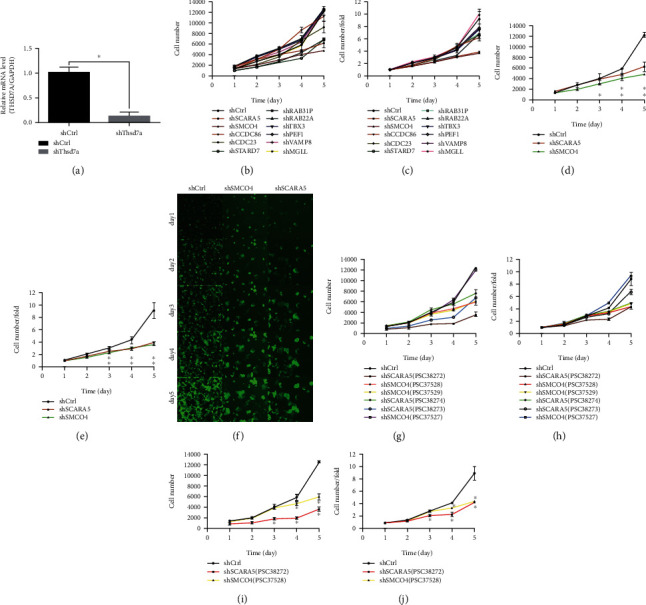
*SCARA5* is the downstream driving gene of *THSD7A*. (a) Expression of *THSD7A* in the Eca109 cells after shRNA-*THSD7A* transfection was detected by qRT-PCR. (b) Cell proliferation curve for 11 candidate genes after shRNA transfection. (c) Cell proliferation fold change curve for 11 candidate genes after shRNA transfection. (d) Cell proliferation curve for the two strongly positive genes. (e) Cell proliferation fold change curve for two strongly positive genes. (f) Fluorescence field pictures of the Eca109 cells from shCtrl, sh-*SCARA5*, and sh-*SMCO4* groups for five days captured by Celigo imaging cytometer. (g) Cell proliferation curve for six interference targets of *SCARA5* and *SMCO4*. (h) Cell proliferation fold change curve for six interference targets of *SCARA5* and *SMCO4*. (i) Cell proliferation curve for the positive interference targets of *SCARA5* and *SMCO4*. (j) Cell proliferation fold change curve for the positive interference targets of *SCARA5* and *SMCO4*. ^∗^*P* < 0.05.

Transfection of shRNA-*THSD7A* into Eca109 resulted in 86.5% knockdown efficiency of the target gene (Figure 1(a)). Further qRT-PCR detection showed that the 37 candidate genes were downregulated to different degrees. We divided candidate genes into three categories according to degree of downregulation: a >50% group, a 40-50% group, and a <40% group. Genes in the <40% group were considered to have no significant change in expression. We then performed functional screening of genes in the >40% group and found that 11 were significantly downregulated (5 genes, >50%; 6 genes, 40%–50%) and expressed as downstream genes of *THSD7A*. Therefore, we carried out further functional screening of these 11 genes, as detailed in Table 2.

### 3.3. Screening Downstream Functional Genes of *THSD7A* via HCS

We designed a mixture of three interference targets per gene with significantly different degrees of downregulation, and then, we transfected the mixtures into the Eca109 cells. Changes in transfected cells' proliferation ability was observed using the Celigo imaging cytometer. We selected those with genes that significantly affected cell proliferation function, and then, we analyzed proliferation ratio of the Eca109 cells in the KD group compared to the control. A ratio of ≥2 was defined as strongly positive, >1.5 as positive, and<1.5 as negative. The results showed two strongly positive genes and nine negative genes, as indicated in Figures 1(b) and 1(c). We separately compared Celigo screening results of two strongly positive genes (Figures 1(d) and 1(e)).

### 3.4. Functional Verification of Single Target of *SCARA5* and *SMCO4*

As two downstream genes of *THSD7A*, *SCARA5* and single-pass membrane protein with coiled-coil domain 4 (SMCO4) showed significant effects on Eca109 proliferation in the Celigo assay with the mixed interference sequence. Next, we separately packaged three interference sequences of each gene into vectors and transfected them into Eca109. The effect of each sequence on the Eca109 proliferation was detected via Celigo cytometer. The results of this single-target experiment showed that of the six interfering targets of the two candidate genes, sh-*SCARA5* (PSC38272)and sh-*SMCO4* (PSC37528) behaved as strongly positive targets, with proliferation rates double or more (Figures 1(f)–1(j) and Table 3). We selected the two most significant sequences for further *in vitro* functional experiments.

### 3.5. Detecting *SCARA5* Expression in the Three ESCC Cell Lines

**Figure 2:**
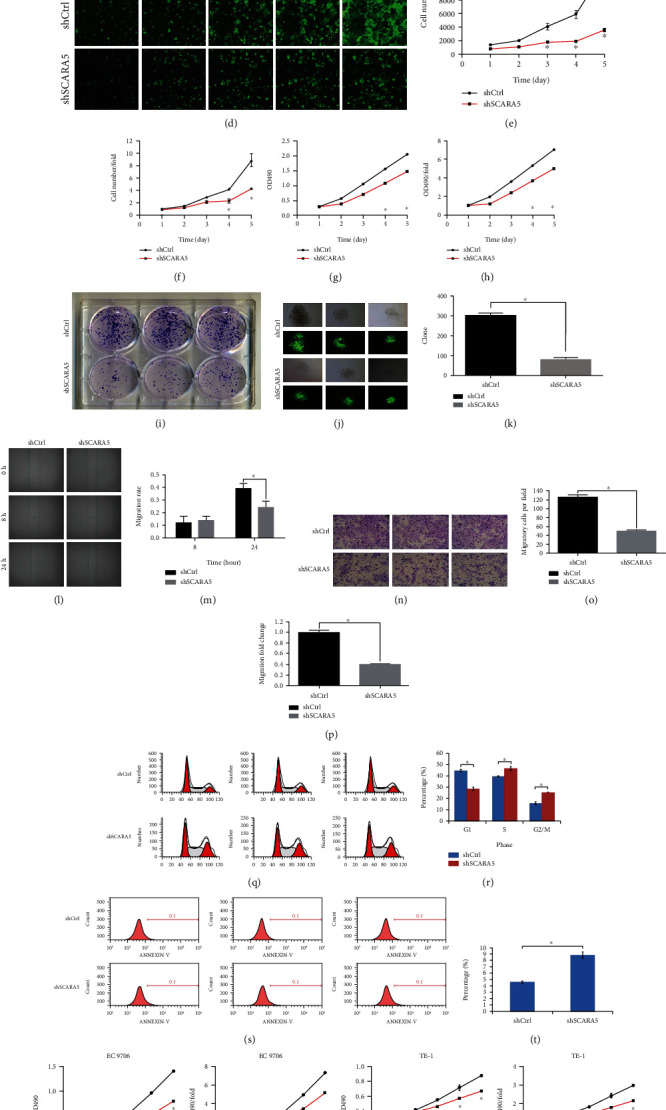
Expression of *SCARA5* in the ESCC cells and its impact on cellular proliferation and migration *in vitro*. (a) Expression of *SCARA5* at mRNA level in the Eca109, EC9706, and TE-1 cells was detected using qRT-PCR. (b) Expression of *SCARA5* at mRNA level in the Eca109 cells of the KD and the control groups was detected using qRT-PCR. (c) Expression of *SCARA5* at protein level in the Eca109 cells of the KD and the control groups was detected using western blot. (d) Fluorescence field pictures of the Eca109 cells from the KD and the control groups were captured by Celigo imaging cytometer for constantly five days. (e–h) Effect of *SCARA5* knockdown on proliferation of the Eca109 cells was evaluated via MTT assay. (i–k) Effect of *SCARA5* knockdown on proliferation of the Eca109 cells was evaluated by clone formation assay. (l and m) Effect of *SCARA5* knockdown on migration of the Eca109 cells was evaluated by wound healing assay. (n–p) Effect of *SCARA5* knockdown on migration of the Eca109 cells was evaluated by cell invasion assay. (q and r) Effect of *SCARA5* knockdown on cell cycle of Eca109 cells was evaluated by FCM. (s and t) Effect of *SCARA5* knockdown on apoptosis of Eca109 cells was evaluated by FCM. (u–x) Effect of *SCARA5* knockdown on proliferation of the EC9706 and TE-1 cells was evaluated via MTT assay. ^∗^*P* < 0.05.

Using qRT-PCR, mRNA levels of *SCARA5* in the ESCC cell lines Eca109, EC9706, and TE-1 were detected. The results showed that *SCARA5* was increased in the Eca109 and EC9706 cells and lowly expressed in the TE-1 cells (Figure 2(a)). Gene expression abundance in the cell was considered high when the *Δ*Ct value ≤12 and low when the *Δ*Ct value ≥16.

### 3.6. Detecting the Interference Efficiency of shRNA-*SCARA5* (PSC38272) Sequence via qRT-PCR and Western Blot

After transfection of sh-*SCARA5* (PSC38272) lentivirus vector into the Eca109 cells for 72 h, qRT-PCR detection found that *SCARA5* at mRNA level in Eca109 of KD group was significantly inhibited (*P* < 0.05), and knockdown efficiency reached 80.9%. Moreover, western blot analysis showed that sh-*SCARA5* (PSC38272) was an effective target due to its knockdown effect on exogenous expression of the *SCARA5* gene (Figures 2(b) and 2(c)).

### 3.7. ShRNA-*SCARA5* Inhibited Proliferation of the Eca109

We used Celigo fixed-point real-time monitoring technology to detect the effect of *SCARA5* on Eca109 cell growth. After transfecting Eca109 cells with shRNA lentivirus for 3 days, we spread the cells into 96-well plate, 2000 cells per well, and continuously monitored them for 5 days using the Celigo cytometer. The results showed that the absolute number of the Eca109 cells and the multiple of change in cell number were obviously reduced in the KD group compared to the control; that is, the proliferation rate of cells was significantly inhibited after *SCARA5* knockdown (*P* < 0.01) (Figures 2(d)–2(f)).

The MTT results showed that for cells in the KD group, the light absorption rate of 490 nm and the multiple of change in light absorption rate decreased significantly after 72 h of transfection compared to the control, and cell proliferation activity was significantly inhibited (*P* < 0.01) (Figures 2(g) and 2(h)).

We conducted a clone formation experiment to detect the effect of *SCARA5* on Eca109 proliferation. After 15 days, we counted the number of cell clones and found the number of Eca109 cell colonies in the KD group significantly reducing compared to the control (*P* < 0.01). The Eca109 clone-forming ability was significantly inhibited after *SCARA5* knockdown (Figures 2(i)–2(k)).

The results of all of the above experiments suggested that *SCARA5* can significantly affect the cell proliferation ability of the Eca109 cells.

### 3.8. ShRNA-SCARA5 Inhibited Migration of the Eca109 Cells

The cell scratch test showed that after transfecting with shRNA-*SCARA5* for 24 h, the scratch width of the Eca109 cells of the KD group was significantly larger than the control, and the difference was statistically significant (*P* < 0.05) (Figures 2(l) and 2(m)). Transwell experiment showed that the number of migrated cells in the KD group was significantly lower than in the control (*P* < 0.05) (Figures 2(n)–2(p)), which showed that *SCARA5* had a significant influence on the migration ability of the Eca109 cells.

### 3.9. ShRNA-*SCARA5* Inhibited Eca109 Cell Cycle in S and G2/M Phases

We detected cell cycle changes via FCM. The results showed that the percentage of cells in S phase and G2/M phase in the KD group was significantly higher than in the control (*P* < 0.05). *SCARA5* might have affected cell proliferation function by acting on cell S phase and G2/M phase (Figures 2(q) and 2(r)).

### 3.10. ShRNA-*SCARA5* Promoted Apoptosis of the Eca109 Cells

After transfecting Eca109 cells with shRNA-*SCARA5* for 48 h, we detected the change in apoptosis rate via FCM. The results showed that the apoptosis rate in the KD group was significantly higher than in the control (*P* < 0.05), meaning that *SCARA5* could significantly affect apoptosis of the Eca109 cells (Figures 2(s) and 2(t)).

### 3.11. ShRNA-*SCARA5* Inhibited Subcutaneous Tumorigenicity of the Eca109 Cells In Nude Mice

**Figure 3:**
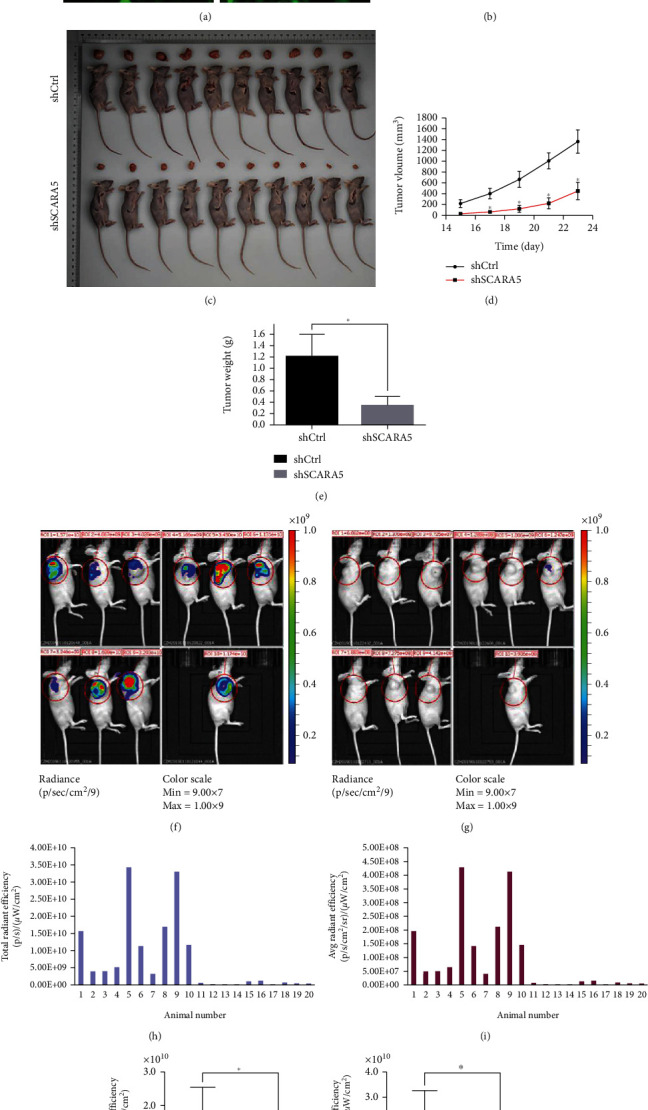
Effect of *SCARA5* knockdown on subcutaneous tumorigenicity of ESCC cells *in vivo*. (a) Pictures of the Eca109 cells form the KD and the control groups were captured by Celigo imaging cytometer after sh-*SCARA5* transfection and before injection. (b) Expression of *SCARA5* at mRNA level in the Eca109 cells was detected using qRT-PCR after shRNA transfection. (c–e) Tumor volume and tumor weight were analyzed between the KD group and the control group to evaluate the effect of *SCARA5* knockdown on subcutaneous tumorigenicity of ESCC cells *in vivo*. (f–k) The vivo imaging assay was applied to evaluate the subcutaneous tumorigenicity of ESCC cells in the KD and the control groups. ^∗^*P* < 0.05.

First, the qRT-PCR test showed that *SCARA5* gene knockdown efficiency in the KD group reached 83.3%. Then, we performed a subcutaneous tumorigenesis experiment using nude mice. *In vivo* imaging showed that both the fluorescence intensity and expression range of the KD group were significantly reduced compared to the control (*P* < 0.05, Figures 3(a) and 3(b)). Tumor-forming experiment showed that the tumor body in the KD was smaller and lighter than the control (*P* < 0.05, Figures 3(c)–3(e)). The *in vivo* imaging experiment showed that the total and average radiant efficiencies of the KD group were weaker than those of the control (Figures 3(f)–3(k)). Therefore, shRNA-*SCARA5* could effectively suppress Eca109 cell subcutaneous tumorigenicity in nude mice. This further verified the cancer-promoting effect of *SCARA5* on the Eca109 cells in our *in vitro* cytology experiments.

### 3.12. Expression Intensity of *SCARA5* in ESCC Tissues Was Remarkably Increased Compared with Normal Mucosal Tissues

**Figure 4:**
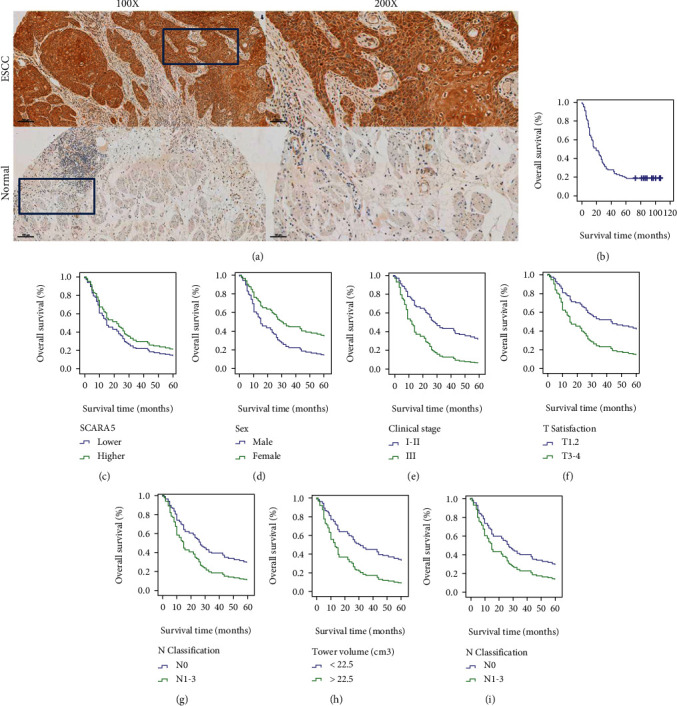
Detection of *SCARA5* expression in ESCC and paired normal tissues and its prognostic significance. (a) Expression of *SCARA5* in ESCC and paired normal tissues was detected using IHC. (b) K-M survival curve for all the patients. (c) *SCARA5* expression was related to the prognoses. (d–h) Sex, clinical stage, T classification, N classification, tumor volume, and longest diameter of the tumor were related to the prognoses according to the univariate prognostic analysis.

We conducted IHC to detect expression levels and features of *SCARA5* in ESCC. As seen in Figure 4(a), only cell membrane and cytoplasm were immunostained positive. We analyzed differences between the 100 cases of ESCC and the 80 paired normal adjacent tissues and found no obvious difference in baseline characteristics among the high and the low *SCARA5* expression groups. The other clinicopathological parameters, including clinical stage, differentiation, tumor location, T/N classification, tumor volume, gross classification, vascular invasion, nerve invasion, and baseline parameters (sex and age), were not with correlation with the expression intensity of *SCARA5* (Table 4).

### 3.13. Expression Intensity of *SCARA5* Was Not Related to Prognosis

The correlation between *SCARA5* intensity and the prognoses of ESCC patients were performed using the K-M survival curves as well as Cox proportional-regression analysis. Medium follow-up duration was 19 months (range, 1–107 months). Patients' 1-, 3-, and 5-year overall survival rates were 63%, 28%, and 19%, respectively (Figure 4(b)). Prognoses did not significantly differ between patients with high and low *SCARA5* expression (Figure 4(c) and Table 5), while the K-M univariate analysis indicated that sex, T/N classification, tumor volume, clinical stage, and longest diameter of tumor were associated with prognosis (Figures 4(d)–4(i) and Table 5); Cox multivariate analysis indicated that tumor volume and T classification were both independent prognostic factors (Table 5).

### 3.14. shRNA-*SCARA5* Inhibited Proliferation of the EC9706 and TE-1 Cells

In the above experiments, *SCARA5* was proven to play cancer-promoting role in the Eca109 cells. However, the authors of all *SCARA5* tumor-related studies published so far believe that *SCARA5* functions as a tumor suppressor gene [15–23]. In order to verify *SCARA5* function in ESCC cells, a MTT assay was performed on ESCC cell lines EC9706 and TE-1. After *SCARA5* was knocked down, the results showed that the EC9706 and TE-1 KD groups significantly decreased in light absorption rate and the multiple of change of 490 nm of cells compared to the control, and the cell proliferation ability was significantly inhibited (*P* < 0.05; Figures 2(u)–2(x)). These experimental results were consistent with those of the Eca109 cells.

### 3.15. Functional Verification of *SCARA5* in Liver Cancer Cells

**Figure 5:**
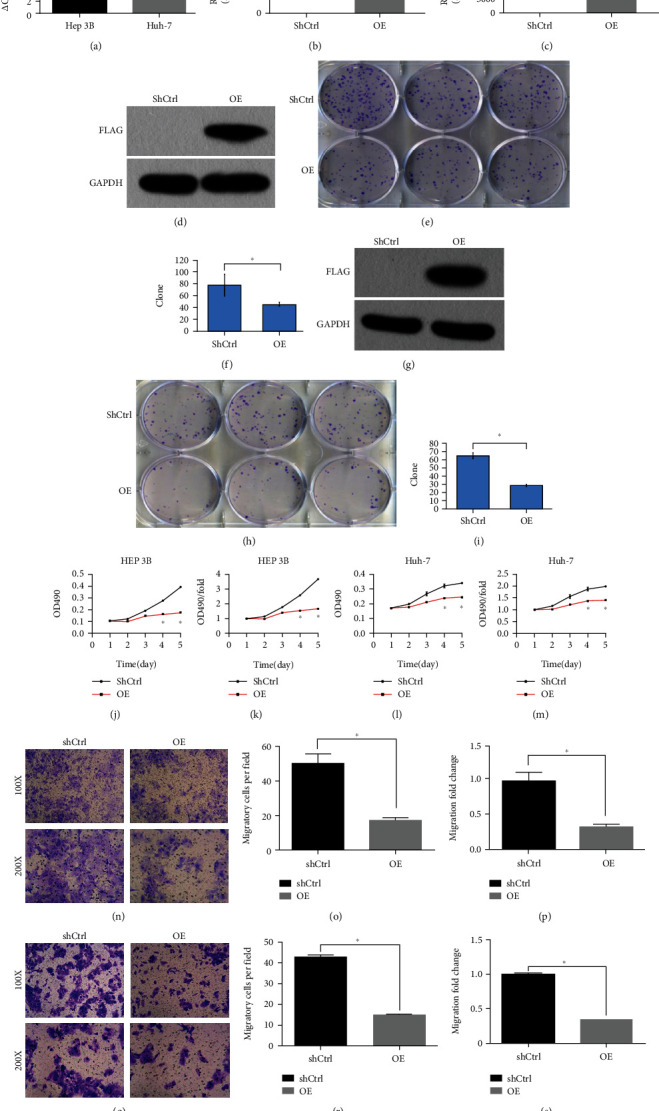
Effect of *SCARA5* overexpression on the proliferation and migration of Hep 3B and Huh-7. (a–c) Expression of *SCARA5* at mRNA level in Hep 3B and Huh-7 cells was detected by qRT-PCR before and after transfection of siRNA. (d and g) Expression of *SCARA5* at protein level in Hep 3B and Huh-7 cells from the OE and the control group was detected by western blot assay. (e, f, h, and i) Effect of *SCARA5* overexpression on the proliferation of Hep 3B and Huh-7 cells was evaluated using clone formation assay. (j–m) Effect of *SCARA5* overexpression on the apoptosis of Hep 3B and Huh-7 cells was evaluated by FCM. (n–s) Effect of *SCARA5* overexpression on the migration of Hep 3B and Huh-7 cells was evaluated by Transwell cell migration assay. ^∗^*P* < 0.05.

Studies published to date on *SCARA5*'s role in tumors have all involved in multiple tumors: the tumor suppressor function of *SCARA5* was first discovered in LC, and the functional studies thereof were comprehensive. In view of the significant differences in *SCARA5* findings in EC, we validated some of the proliferation and migration assays (clone formation, MTT, Transwell, and western blot) in the literature on LC. Clone formation results showed that the number of cell colonies in the Hep 3B and Huh-7 overexpression groups was significantly lower than the control (*P* < 0.05; Figures 5(a)–5(i)). MTT data showed that cell proliferation rate was significantly suppressed in both the Hep 3B and Huh-7 overexpression groups compared to the control (*P* < 0.05; Figures 5(j)–5(m)). Transwell cell migration experiments showed that the number of cell metastases in the Hep 3B and Huh-7 overexpression groups was significantly increased compared to the control (*P* < 0.05; Figures 5(n)–5(s)). Those experiments confirmed that overexpression of *SCARA5* significantly suppressed on Hep 3B and Huh-7 cell proliferation and migration but promoted those in EC and LC.

### 3.16. Exon Sequencing of *SCARA5* in the Three ESCC Cell Lines

**Figure 6:**
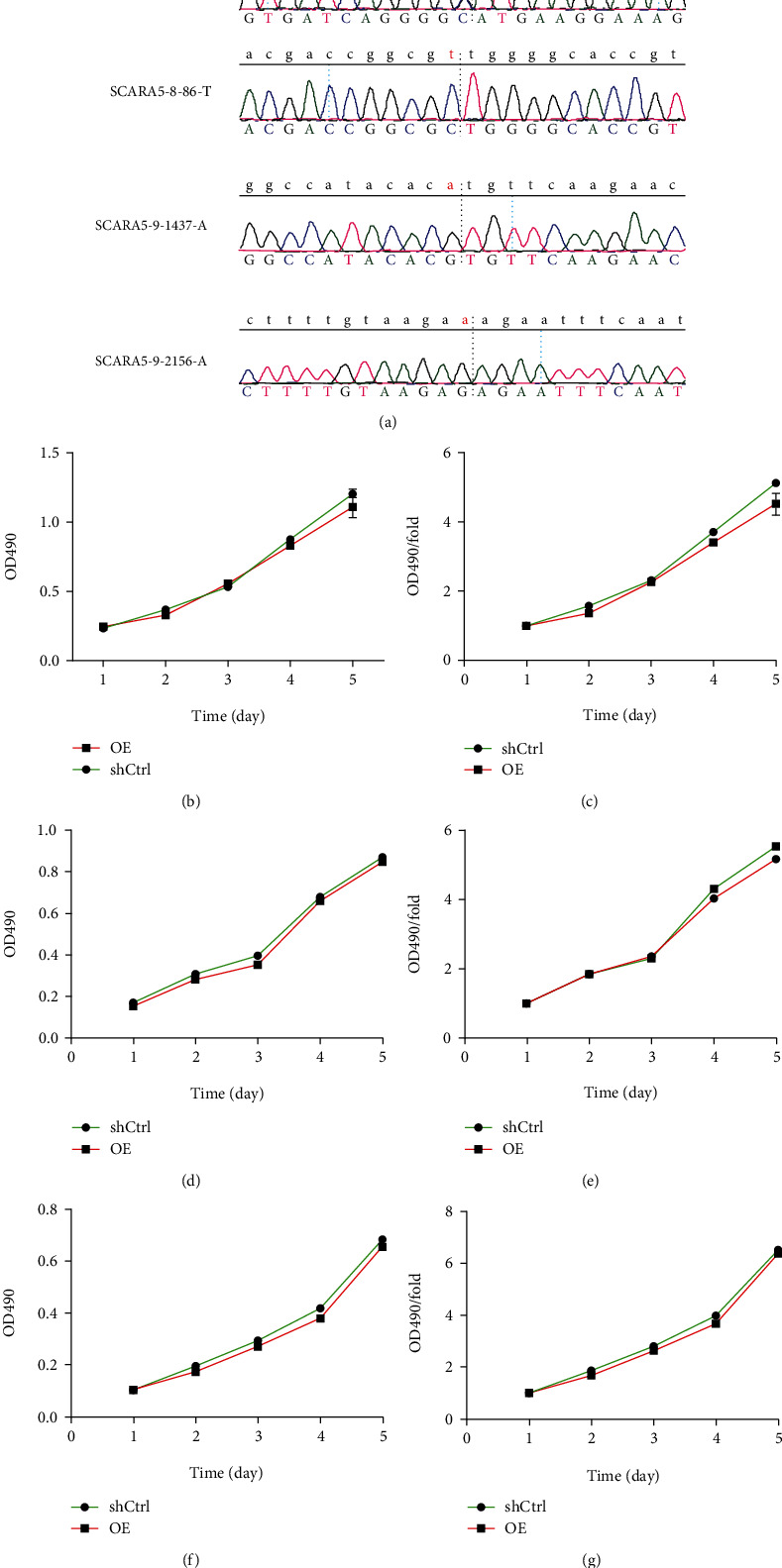
Sequencing of *SCARA5* gene exons in the ESCC cells. (a) 11 exon mutations were found in the three ESCC cell lines, and four of the 11 mutations were identical across all the three cell lines. (b–g) In the Eca109, EC9706, and TE-1 cell lines, no significant changes were observed in cell proliferation in the WT OE group compared with the control group.

To explore why *SCARA5* function in ESCC was opposite to that in other tumors, we performed exon sequencing of the Eca109, EC9706, and TE-1 cells and compared the exon sequences of WT *SCARA5* with those in the PubMed gene database. We found 11 exon mutations in the three cell lines, and 4 of the 11 were identical across all three lines (Figure 6(a) and Table 6). Since *SCARA5* was a proto-oncogene in all three lines, we speculated that one or more of the four common mutations might be responsible for the functional changes in the gene. By comparing codons, we found that two of the four were noncoding mutations, a third was a synonymous mutation, and the last was a missense mutation. The missense mutation was at position 30 of exon 5, resulting in the conversion of methionine to isoleucine. Therefore, we speculated that this site mutation might be the cause of *SCARA5* functional changes in the three ESCC cell lines.

### 3.17. Detecting the Proliferation Function of WT *SCARA5* in the ESCC Cell Lines

We constructed a WT *SCARA5* overexpression vector to determine whether *SCARA5* could play a cancer-promoting role in EC due to mutation, and then, we used a MTT assay to detect proliferation capacity. In Eca109, EC9706, and TE-1 cell lines, we observed no significant changes in the WT overexpression group compared to the control (Figures 6(b)–6(g)). Therefore, our results showed that WT *SCARA5* also played a cancer-promoting role in ESCC cells, negating our previous assumption that mutations had led to functional changes.

### 3.18. Detecting *SMCO4* Proliferation in the ESCC Cell Lines

**Figure 7:**
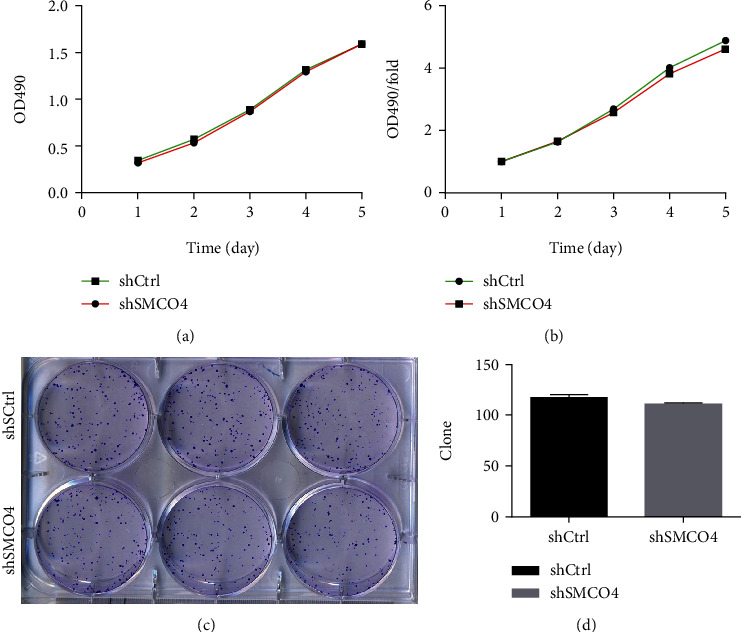
Effect of *SMCO4* knockdown on the proliferation of the Eca109 cells. (a and b) MTT assay was used to evaluate the effect of *SMCO4* knockdown on the proliferation of the Eca109 cells. (c and d) The clone formation assay was applied to observe the effect of *SMCO4* knockdown on the proliferation of the Eca109 cells.

We performed MTT and clone formation experiments on the basis of sh-*SMCO4* knockdown. The MTT assay indicated no significant differences of absorptivity or variation ratio of the Eca109 cells between the KD and the control (Figures 7(a) and 7(b)). Clone formation showed that the number of Eca109 cell colonies was not significantly reduced in the KD and the control. The above experiments indicated that *SMCO4* had no significant influence on Eca109 proliferation (Figures 7(c) and 7(d)).

## 4. Discussion

In this study, in order to clarify *THSD7A*'s cancer-promoting function, mechanism, and suspected downstream genes in ESCC, we performed preliminary screening and validation of THSD7A's downstream driving genes [24]. We are the first to explore the clinical significance, functions, and mechanisms of the downstream gene *SCARA5* of *THSD7A* in ESCC. So far, *SCARA5* has been previously proved as a candidate tumor suppressor for hepatocellular carcinoma (HCC), BC, and other tumors [5, 15, 25–29]. However, critical SCARA5 in ESCC remains unclear. Therefore, we analyzed the clinical significance and function of *SCARA5* in ESCC by using ESCC samples *in vivo* and *in vitro*, and we explored the possible causes of *SCARA5* tumorigenicity. As can be seen from the existing literature, *SCARA5* is associated with conditions that include tumors, inflammation, CVD, hemophilia, retinopathy, bone lesions, connective-tissue disease, and pregnancy [10, 12, 25, 30–33]. Most studies on *SCARA5* are tumor related, involving BC, colorectal cancer (CRC), LC, and other cancers [26, 29, 34]. These studies suggest that *SCARA5* might act a particular role in tumor progression. However, it is worth pondering that most *SCARA5* tumor studies have shown this gene significant effect in tumor inhibition, quite different from the findings of this study.

We began our study by identifying 11 genes, including *SCARA5* and *SMCO4*, as *THSD7A* downstream genes by using qRT-PCR. Then, we used Celigo HCS technology for preliminary screening of mixed targets and secondary screening of single targets and found that *SCARA5* and *SMCO4* might be proto-oncogenes in ESCC [35, 36]. Further functional verification tests confirmed that of the 11 genes involved in ESCC, *SCARA5* was the only oncogene. The reason for the difference between our results and those of previous studies might have been the false-positive rate of HCS [37]. It can be inferred from our results that the upstream oncogene *THSD7A* not only affected the mTOR, extracellular signal-regulated kinase (ERK), and adenosine monophosphate-activated protein kinase (AMPK) signaling pathways but might also upregulate the expression of *SCARA5* to promote cancer in ESCC [24].

In this study, Eca109 proliferation and migration abilities were significantly inhibited after we knocked down *SCARA5* using shRNA. In addition, the results of our mouse subcutaneous-transplantation tumor model experiment were similar to those of our *in vitro* cell proliferation experiment. Proliferation and migration are important markers of malignancy, and *SCARA5* can alter these properties [5, 19]. Our data suggested that *SCARA5* might play a cancer-promoting role in ESCC, which was contrary to the conclusions of most published *SCARA5*-related tumor studies. Therefore, we repeated the proliferation experiments in which *SCARA5* was first found to be an inhibitor in HCC, and we also confirmed the experimental data of tumor inhibition function in LC. The reasons for our results might be as follows: first, *SCARA5* might have been mutated in ESCC, leading to the transformation of a tumor suppressor gene into a tumor promoter gene [38, 39]. Second, the results of multiple tumor tissue samples detected in the ATLAS database show high expression of *SCARA5* in tumors including BC and CRC. Third, EC, especially ESCC, is different from adenoepithelial and other tumors from different tissue sources. Fourth, a recent study on atherosclerosis found that the proliferating and migrating abilities of aortic smooth muscle cells were significantly inhibited after *SCARA5* KD [12]. Moreover, a tumor study showed that elevated ferritin levels in *SCARA5* transport might promote tumor metabolism and growth [21]. The above conclusions are consistent with the results of our study. To some extent, this also indicates the possibility of *SCARA5*-promoting cancer functions. In addition, after *SCARA5* knockdown, the ratio of apoptosis rate to cell cycle arrest in S and G2/M phases was significantly increased, which were also common in tumor progression [5, 27].

To confirm the cancer-promoting effect of *SCARA5* in EC cell lines, based on our multiple proliferation related experiments *in vivo* and *in vitro* on the Eca109cells, we also conducted cell proliferation (MTT) experiments on the EC9706 and TE-1 cell lines. Our findings were consistent with those of the Eca109 experiments, which showed the cancer-promoting effect of *SCARA5*.

Using an ESCC microarray, we found that *SCARA5* was significantly overexpressed in this cancer. However, no significant difference between *SCARA5* expression and other clinicopathological information was found, including TNM stage, classification, and prognosis. This might have been due to the limited sample size; a larger study is needed to explain the clinical significance of *SCARA5*. It should be noted that *SCARA5* expression was significantly different across a variety of tissues, including tumors and stem cells. On the one hand, *SCARA5* expression is downregulated relative to normal tissues in 18 tumors, including esophageal adenocarcinoma (EAC), osteosarcoma, BC, and CRC [22]. On the other hand, it is upregulated in BC stem cells including MDA-MB-231 in triple-negative BC (TNBC) cell lines [23]. More interestingly, contrary to the previous findings, another tumor study found *SCARA5* expression in glioma cells, neuroblasts, and BC [21]. These significant differences reflect that *SCARA5* expression and biological function might be independent in different tissues. Other gene studies have reported differences in gene expression and function in different tumors. DEAD-box RNA helicase 3 (*DDX3*) and ubiquitin-specific peptidase 22 (*USP22*) genes differ significantly in expression and function according to tumor type [38, 39]. The reasons for this difference might include the following: first, genetic mutations might fundamentally alter the function of genes. Second, the gene proteins in different tumors might show different binding states. Third, other factors such as viruses might have an effect [39]. To investigate the first possibility, we found the mutation sites of three ESCC lines by exon sequencing, constructed a WT *SCARA5* overexpression group and a mutant group to compare against one another in a proliferation experiment, and found that WT *SCARA5* also played a role in promoting cancer. Therefore, we concluded that mutations could be ruled out, while the latter two possibilities need to be further explored.

In addition, it should be noted that *SCARA5* metastasis was inconsistent between animal and cellular experiments. The results from clinical samples showed that *SCARA5* was not associated with lymph node metastasis. But the Eca109 cell line was tested for *SCARA5* phenotype after siRNA knockdown, and the results showed that *SCARA5* could promote cell migration, suggesting that the gene might be necessary for *in vitro* migration. One possibility is that this result was due to the limitation of our clinical sample size; another is that the single intervention of the cell experiment was significantly different from the multivariate confounding of clinical samples.

In addition to significant differences between ESCC and matched normal control tissues, we found no significant association between *SCARA5* expression and clinical features. Several limitations of this study must be acknowledged: first, the relatively small sample size of ESCC organizations might have led to biased or inadequate conclusions. Second, considering that the specificity and correctness of primary antibodies might lead to nonreproducibility, all of the primary antibodies should be fully evaluated before experimental use. Third, we used a tissue microarray to detect the phenotype, which might not have reflected the true heterogeneity of *SCARA5* expression in individual tumors. Therefore, our current observations need to be confirmed in more cases.

## 5. Conclusions

We found that *SCARA5* was the downstream gene of *THSD7A* and promoted proliferation and migration of ESCC cells. Moreover, we observed that *SCARA5* was expressed differently between ESCC and normal esophageal tissues, but no significant phenotypic associations were found between *SCARA5* expression and lymph node metastasis, invasion, or prognosis. Our study suggested that *SCARA5* might play an important role in the canceration process and serve as a therapeutic and prognostic target for ESCC.

## Figures and Tables

**Table 1:** Downstream candidate genes of Thsd7a.

Species	Type	Number
Human	CDC23	NM_004661
Human	TBX3	NM_016569
Human	GGTLC1	NM_178311
Human	RAB22A	NM_020673
Human	CCDC117	NM_173510
Human	UHMK1	NM_175866
Human	P3H1	NM_001243246
Human	FAM81A	NM_152450
Human	VAMP8	NM_003761
Human	CETN2	NM_004344
Human	CORO1C	NM_001105237
Human	STK26	NM_016542
Human	PKP3	NM_001303029
Human	RBM8A	NM_005105
Human	YRDC	NM_024640
Human	MYDGF	NM_019107
Human	BEND6	NM_152731
Human	SCARA5	NM_173833
Human	CYB5B	NM_030579
Human	AGPS	NM_003659
Human	CCDC86	NM_024098
Human	GGT2	XM_011530622
Human	MGLL	NM_007283
Human	WSB1	NM_015626
Human	CSGALNACT1	NM_001130518
Human	STARD7	NM_020151
Human	LDLRAD3	NM_174902
Human	MOB1A	NM_001317111
Human	ADCK2	NM_052853
Human	TMOD3	NM_014547
Human	AGGF1	NM_018046
Human	ABHD17C	NM_021214
Human	DNAJC10	NM_018981
Human	PEF1	NM_012392
Human	RAB3IP	NM_175623
Human	NCOA7	NM_001199620
Human	SMCO4	NM_020179

Abbreviations: CDC23: cell division cycle 23; TBX3: T-box transcription factor 3; GGTLC1: gamma-glutamyltransferase light chain 1; RAB22A: Ras-related protein Rab-22A; CCDC117: coiled-coil domain-containing 117; UHMK1: U2AF homology motif (UHM) kinase 1; P3H1: prolyl 3-hydroxylase 1; FAM81A: family with sequence similarity 81 member A; VAMP8: vesicle-associated membrane protein 8; CETN2: centrin-2; CORO1C: coronin-1C; STK26: serine/threonine kinase 26; PKP3: plakophilin 3; RBM8A: RNA-binding motif protein 8A; YRDC: N6-threonylcarbamoylation transferase domain-containing; MYDGF: myeloid-derived growth factor; BEND6: BEN domain-containing 6; SCARA5: scavenger receptor class A member 5; CYB5B: cytochrome b5 type B; AGPS: alkylglycerone phosphate synthase; CCDC86: coiled-coil domain-containing 86; GGT2: gamma-glutamyltransferase 2; MGLL: monoglyceride lipase; WSB1: WD repeat and SOCS box containing 1; CSGALNACT1: chondroitin sulfate N-acetylgalactosaminyltransferase-1; STARD7: StAR-related lipid transfer domain 7; LDLRAD3: low-density lipoprotein receptor class A; MOB1A: MOB kinase activator 1A; ADCK2: AarF domain-containing kinase 2; TMOD3: tropomodulin-3; AGGF1: angiogenic factor with G and FHA domain 1; ABHD17C: alpha/beta hydrolase domain-containing protein 17C; DNAJC10: DnaJ heat shock protein family (Hsp40) member C10; PEF1: penta-EF-hand domain-containing 1; RAB3IP: Rab-3A-interacting protein; NCOA7: nuclear receptor coactivator 7; SMCO4: single-pass membrane protein with coiled-coil domain 4.

**Table 2:** The candidate genes with downregulation ratio of >40% in mRNA after Thsd7a knockdown.

Type	Average (2^-*ΔΔ*Ct^)		Ratio (%)
	NC	shRNA	
CCDC86	1.005	0.477	52.54
CDC23	1.002	0.356	64.47
MGLL	1.002	0.302	69.86
PEF1	1.010	0.479	52.57
RAB22A	1.003	0.412	58.92
RAB3IP	1.002	0.514	48.70
SCARA5	1.001	0.560	44.06
SMCO4	1.001	0.584	41.66
STARD7	1.001	0.550	45.05
TBX3	1.002	0.599	40.22
VAMP8	1.003	0.502	49.95

Abbreviations: CCDC86: coiled-coil domain-containing 86; CDC23: cell division cycle 23; MGLL: monoglyceride lipase; PEF1: penta-EF-hand domain-containing 1; RAB22A: Ras-related protein Rab-22A; RAB3IP: Rab-3A-interacting protein; SCARA5: scavenger receptor class A member 5; SMCO4: single-pass membrane protein with coiled-coil domain 4; STARD7: StAR-related lipid transfer domain 7; TBX3: T-box transcription factor 3; VAMP8: vesicle-associated membrane protein 8.

**Table 3:** The multiplication of cells relative to the first day.

	Type	Day 1	Day 2	Day 3	Day 4	Day 5	Fold change (day5 Ctrl/shRNA)
Average	shCtrl	1	1.43	2.87	4.14	8.87	1
	shSCARA5 (PSC38272)	1	1.29	2.13	2.29	4.28	**2.07**
	shSMCO4 (PSC37528)	1	1.45	2.81	3.39	4.35	**2.04**
	shSMCO4 (PSC37529)	1	1.56	2.85	3.47	4.82	1.84
	shSCARA5 (PSC38274)	1	1.44	2.99	3.65	4.93	1.8
	shSCARA5 (PSC38273)	1	1.44	2.6	3.12	6.79	1.31
	shSMCO4 (PSC37527)	1	1.61	2.86	4.92	9.41	0.94

Bold formatting indicates that a fold change value of ≥2 was defined as strongly positive regarding proliferation ratio. Abbreviations: SCARA5: scavenger receptor class A member 5; SMCO4: single-pass membrane protein with coiled-coil domain 4.

**Table 4:** Clinicopathological significances of SCARA5 expression in esophageal carcinoma patients.

Characteristics	*N*	SCARA5 expression		*X* ^2^	*P* ^a^
		Higher (%)	Lower (%)		
Specimen				55.4	**<0.01**
Adjacent normal tissue	80	13 (16.3)	67 (83.7)		
ESCC	100	72 (72.0)	28 (28.0)		
Sex				0.13	0.72
Male	74	54 (73.0)	20 (27.0)		
Female	26	18 (69.2)	8 (30.8)		
Age (y)				0.25	0.62
<68	54	40 (74.1)	14 (25.9)		
≥68	46	32 (69.6)	14 (30.4)		
Clinical stage				0.59	0.44
I-II	49	37 (75.5)	12 (24.5)		
III	51	35 (68.6)	16 (31.4)		
Tumor location				0.31	0.86^∗^
Upper	4	3 (75.0)	1 (25.0)		
Middle	26	18 (69.2)	8 (30.8)		
Lower	25	19 (76.0)	6 (24.0)		
T classification				0.02	0.88^∗^
T1-2	17	13 (76.5)	4 (23.5)		
T3-4	83	59 (71.1)	24 (28.9)		
N classification				0.51	0.47
N0	45	34 (75.6)	11 (24.4)		
N1-3	55	38 (69.1)	17 (30.9)		
Differentiation				4.75	0.09^∗^
Well	6	6 (100.0)	0 (0.0)		
Moderately	66	48 (72.7)	18 (27.3)		
Poorly	28	18 (64.3)	10 (35.7)		
Number of tumors				0.61	0.44^∗^
1	80	61 (76.3)	19 (23.8)		
2	5	3 (60)	2 (40)		
Longest diameter of tumor (cm)				0.04	0.85
<5	42	32 (76.2)	10 (23.8)		
≥5	43	32 (74.4)	11 (25.6)		
Tumor volume (cm^3^)				0.67	0.41
<22.5	43	34 (79.1)	9 (20.9)		
≥22.5	42	30 (71.4)	12 (28.6)		

Abbreviations: T: tumor; N: lymph node; clinical stage: according to the American Joint Committee on Cancer staging standard; tumor volume: tumor length∗width∗width/2. ^a^Bold type indicates statistical significance. ^∗^Likelihood ratio.

**Table 5:** Univariate and multivariate Cox analysis of esophageal carcinoma specific survival.

Variables	Univariate		Multivariate^a^	
	HR (95% CI)	*P* ^b^	HR (95% CI)	*P* ^b^
SCARA5		0.35		0.96
Lower	1		1	
Higher	0.79 (0.49-1.28)		0.98 (0.56-1.74)	
Sex		**0.03**		0.05
Female	1		1	
Male	1.85 (1.07-3.20)		1.94 (0.99-3.82)	
Age (y)		0.84		—
≥60	1		—	
<60	1.05 (0.68-1.63)		—	
Clinical stage		**<0.01**		—
I-II	1		—	
III	2.43 (1.54-3.86)		—	
Tumor location		0.35		—
Lower	1		—	
Middle	0.78 (0.42-1.45)		—	
Upper	0.37 (0.09-1.57)		—	
T classification		**0.02**		**<0.01**
T1-T2	1		1	
T3-T4	2.24 (1.15-4.37)		2.91 (1.30-6.51)	
N classification		**0.01**		0.11
N0	1		1	
N1-3	1.80 (1.15-2.82)		1.55 (0.90-2.68)	
Differentiation		0.65		—
Well	1		—	
Moderately	1.59 (0.58-4.41)		—	
Poorly	1.45 (0.50-4.20)		—	
Tumor volume (cm^3^)		**<0.01**		**<0.01**
≤22.5	1		1	
>22.5	2.22 (1.36-3.64)		2.30 (1.39-3.80)	
Number of tumors		0.59		—
1	1		—	
2	1.33 (0.48-3.65)		—	
Longest diameter of tumor (cm)		**<0.05**		—
<5	1		—	
≥5	1.63(1.00-2.63)		—	

Abbreviations: T: tumor; N: lymph node; clinical stage: according to the American Joint Committee on Cancer staging standard; tumor volume: tumor length∗width∗width/2. ^a^Adjusted by Cox proportional hazard regression model including all factors, as categorized in Table 4. ^b^Bold type indicates statistical significance.

**Table 6:** Comparison of exon sequence and wild-type sequence of 3 esophageal squamous cell carcinoma cell lines of SCARA5.

Type	SCARA5-1-22-T	SCARA5-1-209-T	SCARA5-4-218-G	SCARA5-5-30-G	SCARA5-8-86-T	SCARA5-8-104-T	SCARA5-9-291-G	SCARA5-9-295-G	SCARA5-9-445-C	SCARA5-9-1437-A	SCARA5-9-2156-A
Eca109	C/T	C/T	A/A	**C/G**	**C/C**	C/C	A/G	G/G	A/C	**G/G**	**G/G**
EC9706	C/T	C/T	A/A	**C/G**	**C/C**	C/T	A/G	G/G	A/C	**G/G**	**G/G**
TE-1	T/T	T/T	G/G	**C/G**	**C/C**	C/T	G/G	A/G	C/C	**G/G**	**G/G**

Bold formatting indicates that 4 of the 11 exon mutations found in the three cell lines were identical across all three lines.

## Data Availability

The datasets used and/or analyzed during the current study are available from the corresponding author on reasonable request.

## Abbreviations

AJCC: American Joint Committee on Cancer
DAB: Diaminobenzidine
DMEM: Dulbecco's Modified Eagle Medium
DDX3: DEAD-box RNA helicase 3
EMT: Epithelial-mesenchymal transition
EC: Esophageal cancer
ESCC: Esophageal squamous cell carcinoma
FAK: Focal adhesion kinase
FBS: Fetal bovine serum
FCM: Flow cytometry
GAPDH: Glyceraldehyde-3-phosphate dehydrogenase
GFP: Green fluorescent protein
HCS: High-content screening
IHC: Immunohistochemistry
MTT: 3-(4,5-Dimethylthiazol-2-yl)-2,5-diphenyltetrazolium bromide
PVDF: Polyvinylidene fluoride
PBS: Phosphate buffer saline
qRT-PCR: Quantitative real-time PCR
SCARA5: Scavenger receptor class A member 5 putative
SDS-PAGE: Sodium dodecyl sulfate-polyacrylamide gel electrophoresis
THSD7A: Thrombospondin type 1 domain-containing 7A
TNM: Tumor node metastasis
USP22: Ubiquitin-specific peptidase 22.

## Supplementary Materials

Supplementary Materials Supplementary File: Table S1: downstream candidate genes of THSD7A.
