# Supplementary material for: Highly Expressing SCARA5 Promotes Proliferation and Migration of Esophageal Squamous Cell Carcinoma
Source: J Immunol Res. 2022 Jun 17;2022:2555647. doi: 10.1155/2022/2555647 (PMC9232322; doi:10.1155/2022/2555647)
Supplement: Supplementary Materials — Supplementary File: Table S1: downstream candidate genes of THSD7A. [file 2555647.f1.pdf]

Table S1 Downstream candidate genes of Thsd7a

Target gene	Forward primer	Reverse primer
CDC23	CATGGCTGCAATAGCAAGAAAG	CGCCTCATTTTTCACTTGTCCT
TBX3	TGAACTCAACAGCCGCTCCTC	CTTCCAAGCCGCTAACCAACC
GGTLC1	GCGTGAGCACTTAATGGCG	GGCAGAGAAGAACTCGGAGG
RAB22A	TTTTGGCCGCTTTGTAGCAC	CAGAAGCTTGGCAAACCACC
CCDC117	GGAGGGTTCTAGAAGGCGTG	GCCACAGCTACTGCCAAAAC
UHMK1	TGTCTGCCTGGAAATAGC	TGGGGATTCTTTTGAGC
P3H1	CGTGGGATTCTCTTCAGGCA	GAAAGGCAAACCAAGGGCG
FAM81A	ATTATCTGCACGGAGTTGGTTG	TGGCTCCACTGTTTTCCTTAAC
VAMP8	TGAGCACTTCAAGACGACATC	ACAATCACGCAGATAAGGACAA
CETN2	AGCGGACTCCTTTGGCTATG	CCCTCATTGCCACCTTCAGT
CORO1C	GTGCCCACATAACGATCAGGT	CAAAATCACCACAGGTTCAGTCA
STK26	ATCTTGTGCAAACCCTGAGTTG	TTCAATCGCCTGATTCCTGCT
PKP3	AAACCTGTCTCGGAACGCTAG	CTGTCCCGCTTCTTCTTGATG
RBM8A	GTACCTAGTGTCTGAGCGGC	TTGTGAATGCTCTCGTCCCC
YRDC	ACAGATACTGCCGTGTGAGAG	GAGGCGTAAAAGGGTTTAGGTC
MYDGF	GCCGCATTTGAAAGGGAAAGT	CAGCTCAGCTTTGAATGCCC
BEND6	CAGAGAGACCCATATTCGGGA	GGCTTTTTATTTTGGCGCACAA
SCARA5	ATCTGCAATCTGCACCCTCC	AACACGTGTATGGCCCAGTT
CYB5B	ATGTCCGGTTCAATGGCGAC	CATGGATCACAAGCCACAGTT
AGPS	GCGCGAGCTACGGGTCT	TTCTTTGGGATGGTGCCCGA
CCDC86	AGCGTCAGCAAGACCTACACC	CCTCCTTATTCTGGGCCAACT
GGT2	CCCAACACTCAGCCTATTTCCA	CCTTCCTGAACGGTGACCTG
MGLL	TCTCGGAATAAGACAGAGGTCG	ATGGAAGACGGAGTTGGTGAC
WSB1	GCCATTTTGACTCCAGTGTCTC	GGAATACGATGCGGACCCAA
CSGALNACT1	GAGGTGAATGCTGGCGTCAAG	AAATCAGAGGCCGTGTAAGG
STARD7	GAGATGAAGCGGTTGGAAGAA	ACTCGGTACTGGTAAAGGTGG
LDLRAD3	CAATGAGTGCAACATACCAGGC	ACTCTTGTCGAAGCAGTCAGG
MOB1A	GACTATTTGATGACTTGGGTTC	GTGCTGGTGATAAATATGGG
ADCK2	CCTGTGTCCAGTTACCAGCA	TTCCAGGGTGAAGGTCTGCAT
TMOD3	CCACCACAGGGCCATTTGATA	TGTAGGGCACATAGTCTTCCC
AGGF1	GCAGCACAGGGGAACTATGT	ATCCGTGCCCTGTGAACTTT
ABHD17C	GGTCAGAGCATTGGGACTGT	TCCTGGTATCCGGAAAAGCC
DNAJC10	GCAGGTTTGACTGTTCCTCTG	GGTCCAAGCGTGGTAACATGA
PEF1	CACTCCACCTCAGGAAAAGGA	TGGCCATGGTGATTCTGACG
RAB3IP	AAGTCGGAAGCAGCTCACAA	TGCAGTTCGTCTCAACTCCC
NCOA7	GCACAGGCGAAACTTTTCTCT	AGCCATAAACCAAATCGTCCC
SMCO4	ACCTCCAAGGACAAGAAGGA	AACACCACGATCAAGAGCAC
GAPDH	TGACTTCAACAGCGACACCCA	CACCCTGTTGCTGTAGCCAAA
